# Post Mortem Examination of the Genito-Urinary Organs

**Published:** 1842-12-03

**Authors:** 


*Post-Mortem Examinations of the Genito-Urinary Organs.*—Civiale conducts his examination of the genito-urinary organs as follows. Incisions on each side of the body are carried from the external margins of the abdominal ring, so as to meet each other at the posterior portion of the anus, and the pubes and ischium are divided in the same direction. The kidneys are detached without dividing the ureters, and, having made an incision through the intestine at the sacrum, the contents of the pelvis are easily removed. Having placed the parts upon a table, the portion of the pubes and ischium which have been sawn through are removed, so as to expose the anterior part of the bladder, the upper surface of the prostate, and the deep portion of the urethra. A catheter is introduced, and, having punctured the bladder at its fundus, an incision is extended through its body and neck, and the membranous portion of the urethra, to the root of the corpus cavernosum. The lateral and lower parts of the urethra are left undivided, for it is here that the chief alterations are found, care being always taken at once to examine the orifices of the seminal canals. In many of the examinations which Civiale has made of persons who have died after retention, or other affection of the urinary organs, he has found great dilatation of these orifices, and large quantities of pus have issued upon pressure of the vesiculæ seminales. The changes in the vesiculæ are those usually found as the result of inflammation, leading to disorganisation; it terminates sometimes by suppuration, but usually by induration, or even ossification.—*Ibid.*

